# Plant medicine metabolite Yulinzhu treating neurological disorder causing polycystic ovary syndrome: a systematic review and a meta-analysis

**DOI:** 10.3389/fphar.2024.1458621

**Published:** 2024-08-15

**Authors:** Beibei Jiao, Ruilin Chen, Si Chen, Jian Zhang, Peijuan Wang, Huaijun Zhou, Weibo Zhao

**Affiliations:** ^1^ Affiliated Hospital of Integrated Traditional Chinese and Western Medicine, Nanjing University of Chinese Medicine, Nanjing, Jiangsu, China; ^2^ Nanjing Drum Tower Hospital Clinical College of Nanjing University of Chinese Medicine, Nanjing, China; ^3^ Department of Gynecology, Wuxi Hospital Affiliated to Nanjing University of Chinese Medicine, Wuxi, Jiangsu, China

**Keywords:** polycystic ovary syndrome, neurological disorders, Yulinzhu, hormonal regulation, neuroprotection

## Abstract

**Background:**

Polycystic Ovary Syndrome (PCOS) is a prevalent endocrine disorder that affects women of reproductive age, characterized by chronic anovulation, hyperandrogenism, and polycystic ovarian morphology. Emerging evidence indicates that neurological disorders play a significant role in the etiology of PCOS, highlighting the complex interplay between the central nervous system (CNS) and ovarian function. Yulinzhu, a traditional Chinese medicine (TCM) formulation, has been traditionally used to regulate menstrual cycles and improve fertility. This study aims to investigate the efficacy and mechanisms of Yulinzhu in treating PCOS induced by neurological disorders.

**Methods:**

An extensive literature search was performed across electronic databases such as PubMed, EMBASE, Cochrane Library, and China National Knowledge Infrastructure (CNKI), covering publications up to 1 June 2024. The review included randomized controlled trials (RCTs) that compared Yulin Zhu with placebo, standard care, or other active treatments in patients with PCOS. Two reviewers independently carried out data extraction and quality assessment. Meta-analyses were conducted using both fixed and random-effects models, with heterogeneity evaluated using the I^2^ statistic.

**Results:**

We screened 891 records and included 6 studi es in the meta-analysis. The meta-analysis revealed that Yulinzhu about effective rate [RR = 1.19, 95% CI (1.10, 1.29), *p* < 0.0001], pregnancy rate [RR = 2.80, 95% CI (1.65, 4.76), *p* < 0.0001] and ovulation rate [RR = 1.33, 95% CI (1.10, 1.62), *p* = 0.04]. Meta-analysis shows the results of follicle estrogen [WMD = 0.69, 95% CI (−0.39, 1.78), *p* = 0.21], luteinizing hormone [WMD = −2.27, 95% CI (−3.86, −0.67), *p* = 0.005], testosterone [WMD = −0.44, 95% CI (−0.64, −0.25), *p* < 0.0001], estradiol [WMD = 16.20, 95% CI (2.74, 29.67), *p* < 0.0001].

**Conclusion:**

This study demonstrates that plant medicine compund Yulinzhu may effectively treats PCOS including hormonal regulation, anti-inflammatory actions, and neuroprotection. We expect further research with larger, well-designed clinical trials to substantiate our conclusions.

## 1 Background

Polycystic Ovary Syndrome (PCOS) is a multifaceted endocrine disorder that affects a significant percentage of women of reproductive age. There is a complex relationship between Polycystic Ovary Syndrome (PCOS) and neurological disorders. Abnormalities in the nervous system can influence the hypothalamic-pituitary-ovarian (HPO) axis and the autonomic nervous system, leading to the development and exacerbation of PCOS. Specific mechanisms include neurotransmitter imbalances and the impact of chronic stress on hormone secretion, which can result in ovarian dysfunction and metabolic disturbances, thereby worsening the symptoms of PCOS. Characterized by hyperandrogenism, ovulatory dysfunction, and polycystic ovarian morphology, PCOS is associated with a range of complications including infertility, metabolic syndrome, and increased cardiovascular risks (Goodarzi et al., 2011). While the etiology of PCOS is multifactorial and not completely understood, it is widely recognized that both genetic and environmental factors contribute to its development (Lizneva et al., 2016). One emerging area of interest is the relationship between neurological disorders and the onset of PCOS. This nexus offers a novel perspective on the pathophysiology of PCOS and potential therapeutic approaches, such as the use of traditional Chinese medicine (TCM) formulations like Yulinzhu (Macut et al., 2017). Neurological disorders, including stress-related conditions and abnormalities in central nervous system regulation, have been implicated in the development and exacerbation of PCOS (Stener-Victorin et al., 2020). The hypothalamic-pituitary-gonadal (HPG) axis plays a crucial role in regulating reproductive function, and disturbances in this axis due to neurological disorders can lead to hormonal imbalances characteristic of PCOS (Chang and Dunaif, 2021). Chronic stress, for example, can alter the secretion of gonadotropin-releasing hormone (GnRH), leading to dysregulation of luteinizing hormone (LH) and follicle-stimulating hormone (FSH) secretion, ultimately affecting ovarian function. Additionally, the autonomic nervous system and neurotransmitter imbalances are believed to influence insulin resistance and androgen production, both of which are key metabolites in the pathogenesis of PCOS (Ganie et al., 2019).

Yulinzhu, a traditional Chinese medicinal formulation, has been used for centuries in the treatment of various gynecological disorders. Its application in modern medicine, particularly in the treatment of PCOS, is based on its multifaceted pharmacological effects (Rothenberg et al., 2018). Yulinzhu is composed of a variety of botanical drugs that work synergistically to regulate hormonal balance, improve insulin sensitivity, and reduce inflammation. Studies have shown that the metabolites in Yulinzhu can modulate the HPG axis, enhance ovarian function, and alleviate the metabolic disturbances associated with PCOS (Islam et al., 2022).

The integration of TCM in the treatment of PCOS provides a holistic approach that targets multiple aspects of the disorder. Traditional Chinese medicine emphasizes the balance of the body’s systems and the harmonization of internal energies, which aligns well with the multifactorial nature of PCOS (VanHise et al., 2023). The use of Yulinzhu aims to address both the symptoms and underlying causes of PCOS, offering a potential alternative or complementary therapy to conventional treatments. Research into the efficacy of Yulinzhu for PCOS, particularly when the condition is precipitated by neurological disorders, is still in its early stages (Fan et al., 2023). However, preliminary studies and clinical observations suggest promising outcomes (Wang et al., 2022).

Paeonia lactiflora, or White Peony root, is another vital extract in Yulinzhu. It is used for its properties in nourishing the blood, soothing the liver, and relieving pain, which are particularly beneficial in managing the symptoms of PCOS, such as irregular menstruation and dysmenorrhea (Rebar and Keator, 2024). Similarly, Ligusticum chuanxiong, known as Chuanxiong, is included for its effectiveness in promoting blood circulation and alleviating pain, thereby addressing the stagnation often associated with PCOS (Liu et al., 2020). Dioscorea opposita, or Chinese Yam, is included in Yulinzhu for its ability to strengthen the spleen and stomach, nourish the kidney, and consolidate essence, which supports metabolic health and hormonal regulation (McGowan and Quinlivan, 2019). Poria cocos, derived from a fungus, is another metabolite that aids in dispelling dampness, promoting urination, and enhancing spleen function, thereby contributing to the management of metabolic syndrome and insulin resistance commonly seen in PCOS patients (Abruzzese et al., 2023). Alisma orientale, or Alisma, is utilized for its diuretic properties, helping to eliminate excess fluids and clear heat, which can assist in reducing inflammation and metabolic disturbances (Soares Júnior et al., 1992; Han et al., 2023). Paeonia suffruticosa, also known as Moutan Cortex, is included for its ability to clear heat and cool the blood, promoting blood circulation and reducing stasis, which are essential in addressing the hormonal imbalances in PCOS (McGowan and Quinlivan, 2019). Leonurus japonicus, or Motherwort, is another botanical drug in Yulinzhu that is known for its ability to regulate menstruation and promote blood circulation, helping to alleviate the symptoms of PCOS (Pei et al., 2021). *Equus asinus*, or donkey-hide gelatin (Ejiao), although not a plant extract, is included for its potent blood-nourishing and hemostatic effects, complementing the overall formulation by improving blood quality and circulation (Murri et al., 2014).

Yulinzhu’s efficacy in treating PCOS is deeply rooted in the synergistic effects of its plant extracts. Each herb contributes specific therapeutic properties, ranging from blood nourishment and circulation enhancement to hormonal regulation and metabolic support. The integration of these diverse medicinal plants into a single formulation exemplifies the holistic approach of traditional Chinese medicine in managing complex endocrine disorders like PCOS (Papalou et al., 2016).

The connection between neurological disorders and PCOS also underscores the importance of a multidisciplinary approach in managing the condition (Presswala and De Souza, 2023). Neurologists, endocrinologists, and gynecologists must collaborate to develop comprehensive treatment plans that address the complex interplay between the nervous system and reproductive health (Andrade et al., 1992). The use of TCM, and specifically Yulinzhu, can be an integral part of this approach, providing benefits that extend beyond symptom management to address the root causes of the disorder (Wenli, 2020). In this meta-analysis we assessed the efficacy and safety of Yulinzhu about treating neurological disorder causing polycystic ovary syndrome.

## 2 Methods

### 2.1 Study objectives

The primary objective of this study is to systematically evaluate the clinical efficacy of Yulinzhu in treating Polycystic Ovary Syndrome (PCOS), thereby providing evidence-based support and reference for its clinical application.

### 2.2 Composition and taxonomic validation of compound Yulinzhu

The composition of Compound Yulinzhu has been taxonomically validated using resources such as the Medicinal Plant Names Services (MPNS) and Plants of the World Online (POWO). The full species names, including the authority, family, and pharmacopeial names, are provided below.

### 2.3 Literature search

A comprehensive literature search was conducted using multiple databases to ensure a thorough collection of relevant studies. The databases searched included:

(a) PubMed: A free resource developed and maintained by the National Center for Biotechnology Information (NCBI) at the U.S. National Library of Medicine (NLM). It provides access to a vast collection of biomedical and life sciences literature.
(b) Web of Science: A multidisciplinary database that includes citation data from various scientific disciplines, offering access to multiple research databases that allow for in-depth exploration of specialized sub-fields within an academic or scientific discipline. It included the following databases.
(c) Cochrane Library: A collection of high-quality, independent evidence to inform healthcare decision-making. It includes the Cochrane Database of Systematic Reviews and the Cochrane Central Register of Controlled Trials.
(d) SinoMed: A comprehensive database covering a wide range of Chinese medical journals, providing valuable access to literature published in China.
(e) China National Knowledge Infrastructure (CNKI): A key national information construction project of China, providing comprehensive access to a wide array of Chinese academic journals and dissertations.
(f) VIP Database: A significant Chinese database that offers extensive coverage of Chinese academic journals in various fields.
(g) Wanfang Database: Another essential Chinese database providing access to a large collection of academic journals, dissertations, conference proceedings, and other scholarly resources.

The search strategy employed a combination of relevant keywords and medical subject headings (MeSH) terms such as “Yulinzhu,” “Polycystic Ovary Syndrome,” and “PCOS.” We included all relevant studies published from the inception of these databases until 1 June 2024 (The details are in the supplementary document).

### 2.4 Inclusion criteria

This review included randomized controlled trials (RCTs), whether blinded or not, that were published in Chinese or international journals. The participants were women of reproductive age diagnosed with PCOS based on the 2018 Chinese Medical Association guidelines for PCOS diagnosis, which include:

(a) Infrequent or absent menstrual periods or irregular uterine bleeding.
(b) At least one of the following:
  (1) Clinical hyperandrogenism (e.g., acne, hirsutism) or biochemical hyperandrogenemia (serum testosterone ≥2.6 nmol/L, typically not exceeding twice the upper limit of normal).
  (2) Polycystic ovarian morphology (PCOM) on ultrasound, defined as ovarian volume ≥10 mL and/or the presence of ≥12 follicles of 2–9 mm diameter in one or both ovaries.
  (3) Exclusion of other disorders that could cause hyperandrogenism or ovulatory dysfunction, such as adrenal hyperplasia or Cushing’s syndrome.

### 2.5 Exclusion criteria

Studies were excluded if they:

(a) Had incomplete, biased, erroneous, or unobtainable data.
(b) Were duplicate publications.
(c) Were reviews, expert opinions, conference papers, animal experiments, case reports, or non-RCT clinical trials.
(d) Did not clearly specify the intervention measures, or the intervention did not primarily involve Yulinzhu.
(e) Evaluated outcomes unrelated to PCOS efficacy.

### 2.6 Interventions

The control group received standard Western medical treatment, while the experimental group received Yulinzhu in addition to the control treatment or Yulinzhu-based herbal modifications. The formulation of Yulinzhu was limited to decoctions, with no restrictions on the origin, dosage, administration method, or treatment duration of the botanical drugs used.

### 2.7 Primary outcome measure

(a) Overall Clinical Efficacy: This is defined as the combined rate of significant and effective responses. It is a composite measure that includes improvements in clinical symptoms and laboratory findings, indicating the overall effectiveness of the treatment.
(b) Safety: Whether Yulinzhu will bring adverse event.

### 2.8 Secondary outcome measures

(a) Pregnancy Rate: The proportion of women who achieved pregnancy during the study period.
(b) Ovulation Rate: The proportion of women who experienced ovulation as determined by clinical or laboratory assessments.
(c) Hormone Levels: Changes in key hormonal indicators, including:
  Luteinizing Hormone (LH): A hormone that plays a crucial role in regulating the menstrual cycle and ovulation.
  Follicle-Stimulating Hormone (FSH): A hormone essential for ovarian follicle growth and development.
  Testosterone: A hormone often elevated in women with PCOS, contributing to symptoms such as hirsutism and acne.
  Estradiol: A form of estrogen that is important for reproductive and menstrual function.

These outcome measures were chosen to provide a comprehensive assessment of the efficacy of Yulinzhu in treating PCOS, addressing both clinical and biochemical aspects of the condition.

### 2.9 Data extraction

Relevant literature on the treatment of PCOS with Yulinzhu was identified from the databases. The NoteExpress 3.7.0 software was used to manage the literature, excluding duplicates. Titles and abstracts were screened to exclude irrelevant studies, focusing on those that involved RCTs with Yulinzhu. Full texts of potentially eligible studies were then reviewed to confirm inclusion criteria. Extracted data included:

1. Basic information (publication date, first author).
2. Sample sizes of treatment and control groups.
3. Intervention details for both groups.
4. Treatment duration.
5. Clinical outcomes (e.g., efficacy rates, pregnancy rates).

### 2.10 Risk of bias and quality assessment

The Cochrane Collaboration’s “Risk of Bias” tool was used to assess bias across seven domains: random sequence generation, allocation concealment, blinding of participants and personnel, blinding of outcome assessment, completeness of outcome data, selective reporting, and other potential biases. Each domain was rated as “low risk,” “high risk,” or “unclear risk,” with justifications provided for each rating.

### 2.11 Statistical analysis

Data analysis was performed using RevMan 5.4 software recommended by the Cochrane Collaboration. Continuous variables were analyzed using mean difference (MD) or standardized mean difference (SMD) to describe effect sizes, while dichotomous variables were analyzed using relative risk (RR), with 95% confidence intervals (CI). Heterogeneity was assessed using the Chi-square test and I^2^ statistic. If *p* > 0.10 and I^2^ ≤ 50%, a fixed-effect model was used; otherwise, a random-effects model was employed. Sensitivity analysis or subgroup analysis was conducted to explore sources of heterogeneity. Funnel plots were used to assess publication bias, with symmetrical plots indicating minimal bias and asymmetrical plots indicating potential bias.

### 2.12 Heterogeneity analysis

The meta-analysis revealed some heterogeneity in the intervention protocols and outcome measures across the included studies. To explore the sources of heterogeneity, we conducted subgroup analyses and sensitivity analyses. Subgroup analyses will be performed based on different intervention protocols (e.g., dosage, duration of treatment) and specific outcome measures if it can reduce the heterogeneity. These analyses helped identify variations that contributed to heterogeneity and provided a clearer understanding of the overall effects. Sensitivity analyses were conducted by excluding studies with high risk of bias or significantly different protocols. The results of these analyses indicated that the main findings were robust and not significantly influenced by the exclusion of these studies.

### 2.13 Safety assessment

In addition to efficacy, we evaluated the safety of Yulinzhu treatment by assessing the incidence of adverse events reported in the included studies. Safety data were extracted and analyzed to provide a comprehensive overview of the potential risks associated with Yulinzhu treatment.

## 3 Results

### 3.1 Literature retrieval process

**FIGURE 1:**
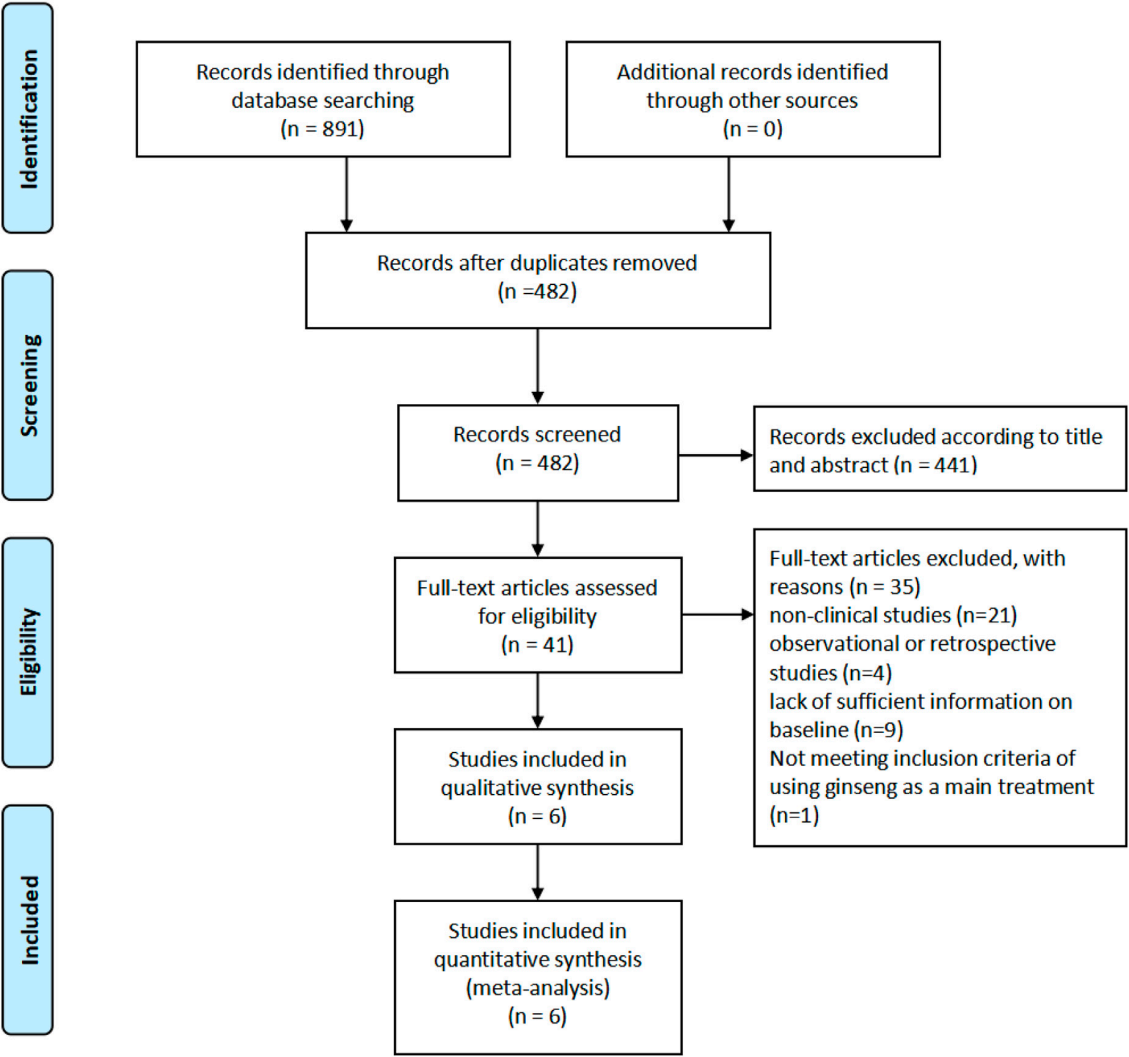
Literature retrieval process.

We conducted a systematic literature screening and analysis process. Initially, 891 records were identified through database searching, with no additional records identified from other sources. After removing duplicates, the number of records was reduced to 482. Subsequently, these records were screened by title and abstract, resulting in the exclusion of 441 records, leaving 41 records for full-text assessment. Upon full-text review, 35 articles were excluded for various reasons: 21 were non-clinical studies, 4 were observational or retrospective studies, 9 lacked sufficient baseline information, and 1 did not meet the inclusion criteria of using ginseng as the main treatment. Ultimately, 6 studies were included in the qualitative synthesis, all of which were also included in the quantitative synthesis (meta-analysis) (Figure 1).

### 3.2 Characteristic of include literature

The table summarizes six studies, each comparing a control group (Con.) and an experimental group (Exp.), both consisting of the same number of participants. In these studies, the control group received regular treatment, while the experimental group received a combination of Yulinzhu and regular treatment. The duration of the interventions varied slightly, with most studies lasting 3 months, except for He 2020, which lasted 12 weeks. The studies included in the table are He 2020, Li 2018, Li 2019, Lou 2019, Sun 2015, and Wei 2019 (Tables 1–3).

**TABLE 1:** Composition and taxonomic validation of compound Yulinzhu.

Ingredient	Family	e	Quantity (g)	Function
Paeonia lactiflora Pall. [Paeoniaceae; Radix Paeoniae Alba]	Paeoniaceae	Radix Paeoniae Alba	15	Nourishing blood, soothing the liver
Ligusticum chuanxiong Hort. [Apiaceae; Rhizoma Chuanxiong]	Apiaceae	Rhizoma Chuanxiong	10	Promoting blood circulation, alleviating pain
Dioscorea opposita Thunb. [Dioscoreaceae; Rhizoma Dioscoreae]	Dioscoreaceae	Rhizoma Dioscoreae	15	Strengthening spleen, nourishing kidney
Poria cocos (Schw.) Wolf [Polyporaceae; Sclerotium Poriae Cocos]	Polyporaceae	Sclerotium Poriae Cocos	10	Enhancing spleen function, dispelling dampness
Alisma orientale (Sam.) Juz. [Alismataceae; Rhizoma Alismatis]	Alismataceae	Rhizoma Alismatis	10	Diuretic properties, reducing inflammation
Paeonia suffruticosa Andr. [Paeoniaceae; Cortex Moutan]	Paeoniaceae	Cortex Moutan	10	Clearing heat, promoting blood circulation

**TABLE 2:** Characteristic of include literature.

Study	No. of Con	No. of Exp	Intervention of Con	Intervention of Exp	Period	Result
He 2020 (Hong, 2018)	54	54	Yulinzhu + regular treatment	regular treatment	12 weeks	a.b.c.d.e.f.g
Li 2018 (Hong, 2019)	45	45	Yulinzhu + regular treatment	regular treatment	3 months	a.b.c.d.e.f.g
Li 2019 (Xueli, 2019)	52	52	Yulinzhu + regular treatment	regular treatment	3 months	a.b.c.d.e.f.g
Lou 2019 (Sun et al., 2015)	37	37	Yulinzhu + regular treatment	regular treatment	3 months	a.b.c.d.e.f
Sun 2015 (Wei, 2019)	30	30	Yulinzhu + regular treatment	regular treatment	3 months	a
Wei 2019 (Wu et al., 2017)	30	30	Yulinzhu + regular treatment	regular treatment	3 months	a.b.c.d.e.f.g

Note: (a) Total effective rate, (b) Ovulation rate, (c) Pregnancy rate, (d) Luteinizing hormone (LH), (e) Follicle stimulation (FSH), (f) estradiol (E2), (g) testosterone (T).

**TABLE 3:** Composition, processing, and extr action of compound Yulinzhu.

Study	Composition Consistency	Processing and extraction details reported	Preparation method
He 2020	Yes	Yes	Standardized decoction
Li 2018	Yes	Yes	Standardized decoction
Li 2019	Yes	Yes	Standardized decoction
Lou 2019	Yes	Yes	Standardized decoction
Sun 2015	Yes	Yes	Standardized decoction
Wei 2019	Yes	Yes	Standardized decoction

### 3.3 Risk of bias about included studies

**FIGURE 2:**
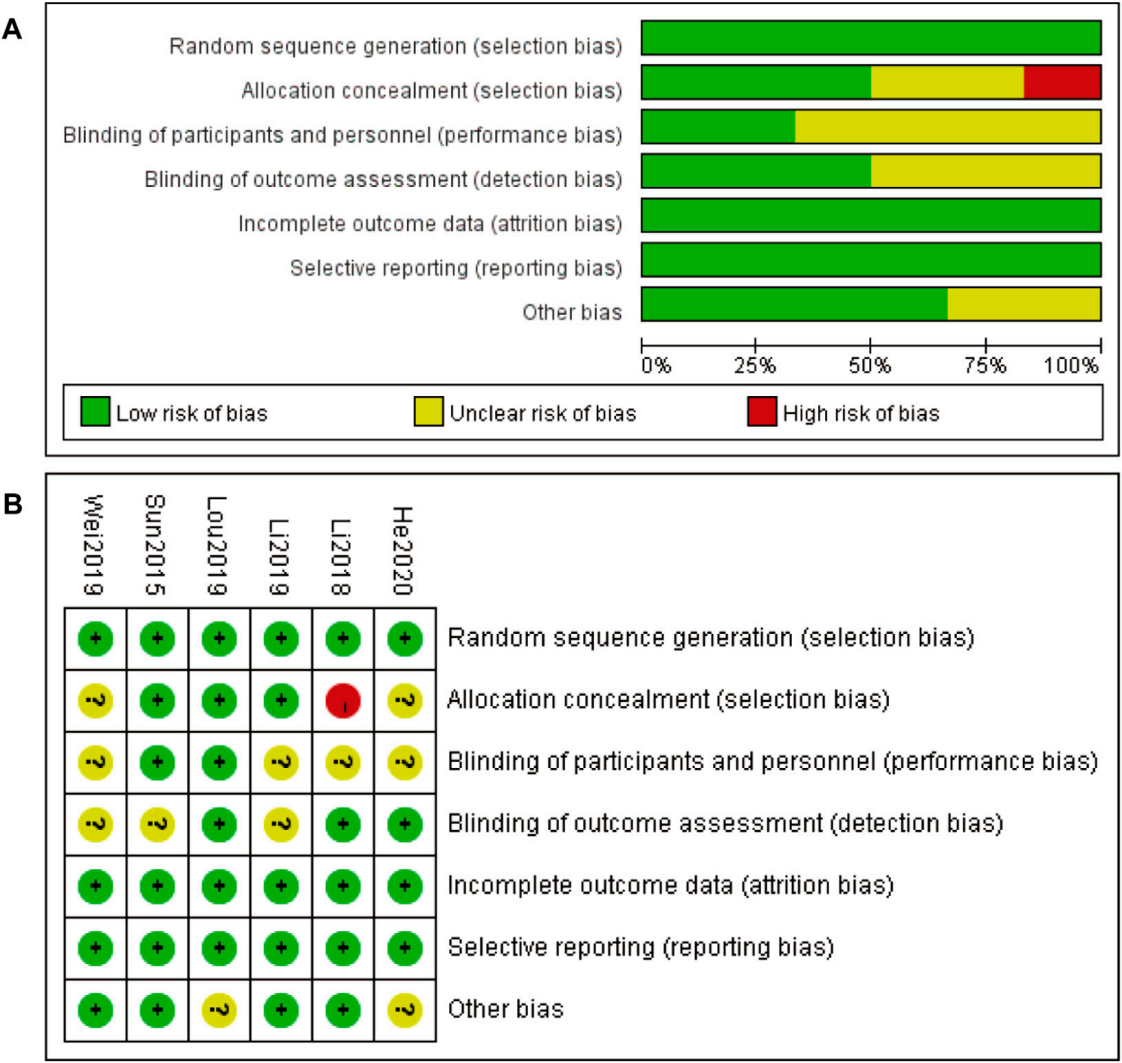
Risk of bias **(A)** Risk of bias graph 3

The risk of bias assessment for the included studies is presented in two parts. Figure 2A provides an overall summary of the risk of bias across various domains, including random sequence generation, allocation concealment, blinding of participants and personnel, blinding of outcome assessment, incomplete outcome data, selective reporting, and other biases. Most domains exhibit a low risk of bias, indicated by the green bars, with some domains showing an unclear risk of bias, represented by the yellow bars. Allocation concealment shows a small proportion of high risk of bias, indicated by the red bar. Figure 2B details the risk of bias for each included study individually. Each study is assessed across the same domains as in part A, with green circles indicating low risk of bias, yellow circles indicating unclear risk of bias, and red circles indicating high risk of bias. Most studies demonstrate a low risk of bias across most domains, with a few instances of unclear risk and one instance of high risk of bias in allocation concealment for the study Li 2018. Overall, the risk of bias assessment suggests that the included studies are generally of high quality, with minimal risks that could affect the validity of the findings (Figure 2).

### 3.4 Effective rate

**FIGURE 3:**
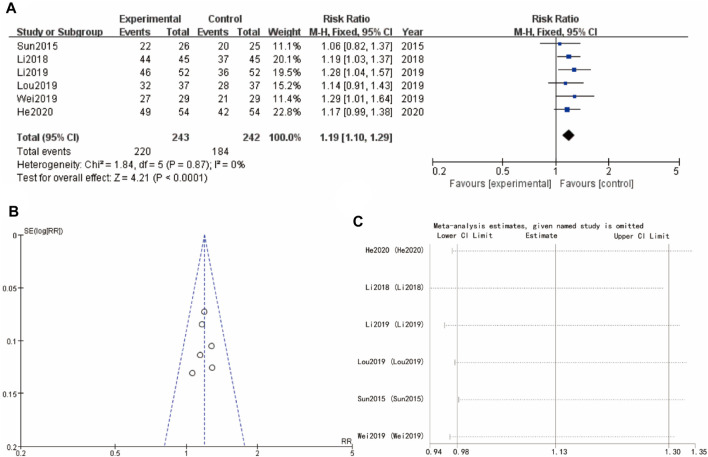
Effective rate of Yulinzhu treatment **(A)** forest plot of effective rate **(B)** funnel plot of effective rate **(C)** Sensitivity analysis.

The results of the meta-analysis shows that a forest plot comparing the risk ratios (RR) for the experimental and control groups across multiple studies (Sun 2015, Li 2018, Li 2019, Lou 2019, and He 2020). The overall effect size indicates a significant benefit of the experimental intervention (Yulinzhu + regular treatment) compared to the control (regular treatment alone), with a combined RR of 1.19 [1.10, 1.29] and a high level of confidence (*p* < 0.0001). The accompanying funnel plot assesses the potential for publication bias, showing a symmetrical distribution of studies. The results of heterogeneity analysis I^2^ and sensitivity analysis show that the differences between groups are very small (Figure 3).

### 3.5 Pregnancy rate

**FIGURE 4:**
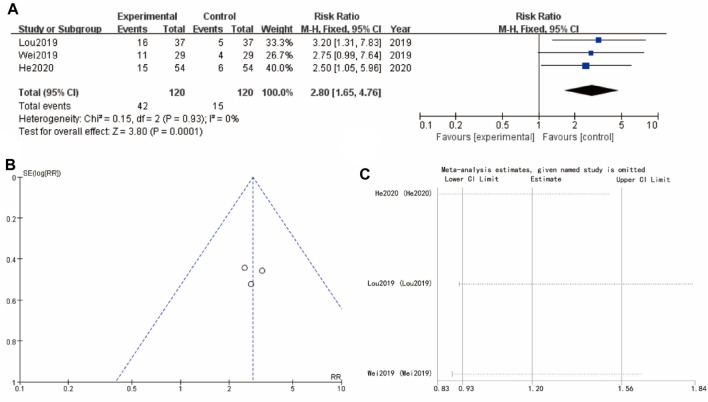
Pregnancy rate of Yulinzhu treatment **(A)** forest plot of pregnancy rate **(B)** funnel plot of pregnancy rate **(C)** Sensitivity analysis.

Figure 4A focuses on pregnancy rate about studies (Lou 2019, Wei 2019, and He 2020), showing a significant improvement in the experimental group with a combined RR of 2.80 [1.65, 4.76] (*p* < 0.0001). The heterogeneity among these studies is low, indicating consistent results across the studies. The funnel plot (Figure 4B) for this subgroup also suggests minimal publication bias. Sensitivity analysis show that the differences between groups are very small (Figure 4C) (Figure 4).

### 3.6 Ovulation rate

**FIGURE 5:**
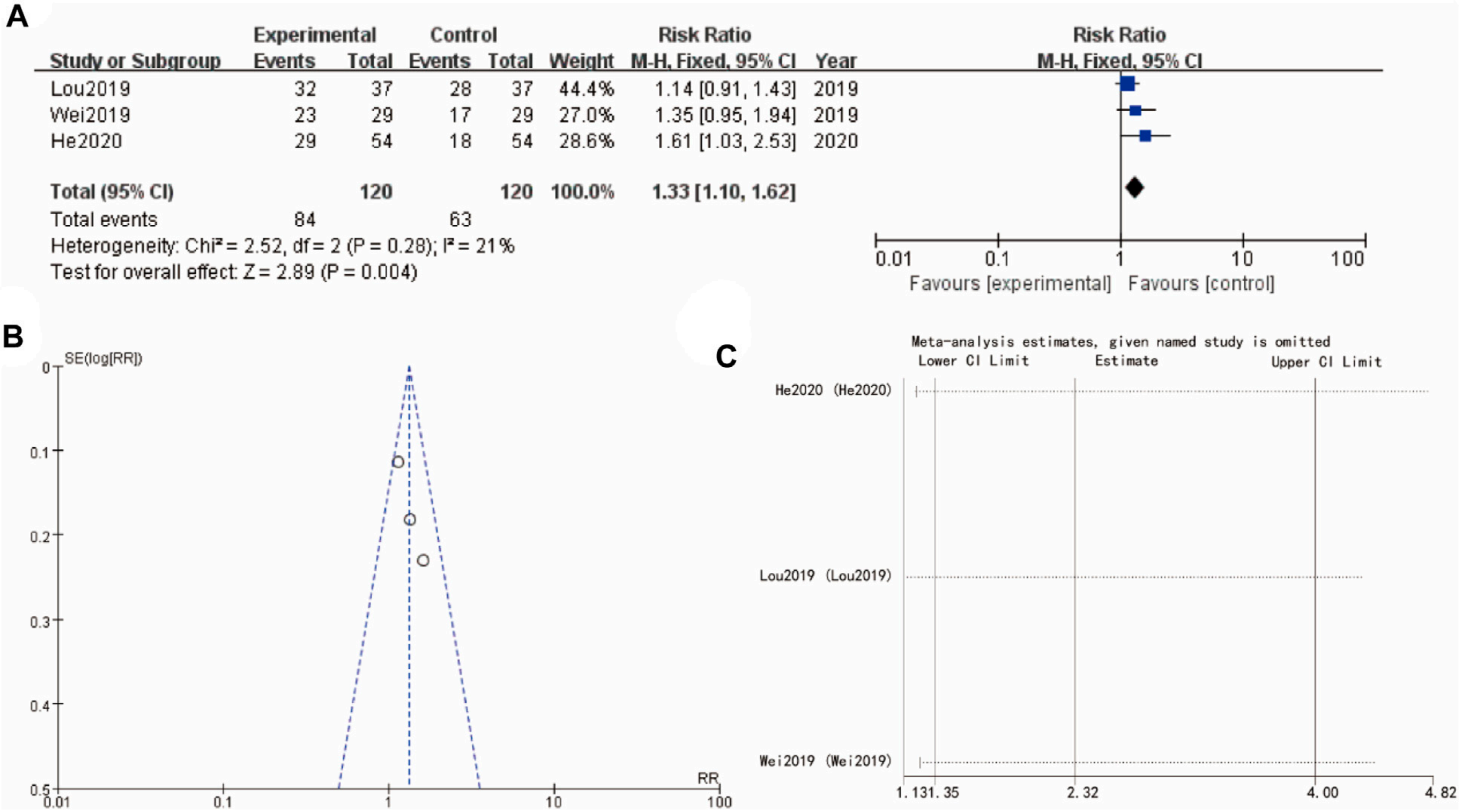
Ovulation rate of Yulinzhu treatment **(A)** forest plot of ovulation rate **(B)** funnel plot of ovulation rate **(C)** Sensitivity analysis.

Figure 5A examines ovulation rate from studies (Lou 2019, Wei 2019, and He 2020), with a combined RR of 1.33 [1.10, 1.62] (*p* = 0.004), indicating a favorable effect of the experimental intervention. The heterogeneity is slightly higher but still acceptable, and the funnel plot (Figure 5B) does not show significant asymmetry, implying low risk of publication bias. Sensitivity analysis show that the differences between groups are very small (Figure 5C).

### 3.7 Follicle-stimulating hormone

**FIGURE 6:**
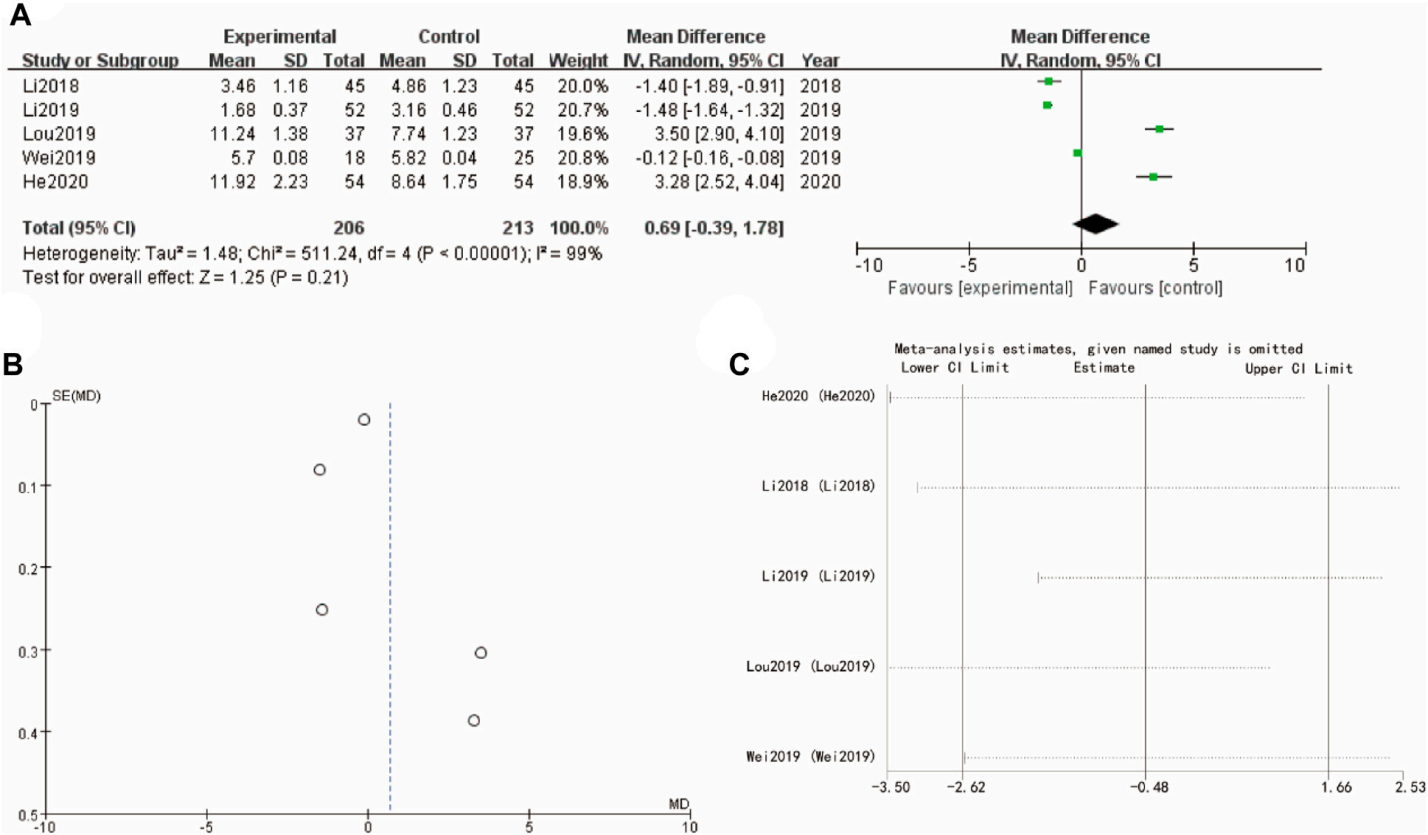
F Follicle-Stimulating Hormone of Yulinzhu treatment **(A)** forest plot of follicle-Stimulating Hormone **(B)** funnel plot of follicle-Stimulating Hormone **(C)** Sensitivity analysis.

The results presented in Figure 6 evaluates the mean differences of follicle-stimulating hormone between the experimental group (Yulinzhu + regular treatment) and the control group (regular treatment alone) across follicular estrogen. In Figure 6A, the forest plot compares the mean differences for several studies (Li 2018, Lou 2019, Wei 2019, He 2020), showing WMD = 0.69 95% CI [−0.39, 1.78] with a p-value of 0.21. Funnel plot in Figure 6B assesses the potential for publication bias, showing a somewhat symmetrical distribution. Sensitivity analysis show that the differences between groups are very small (Figure 6C).

### 3.8 Luteinizing hormone

**FIGURE 7:**
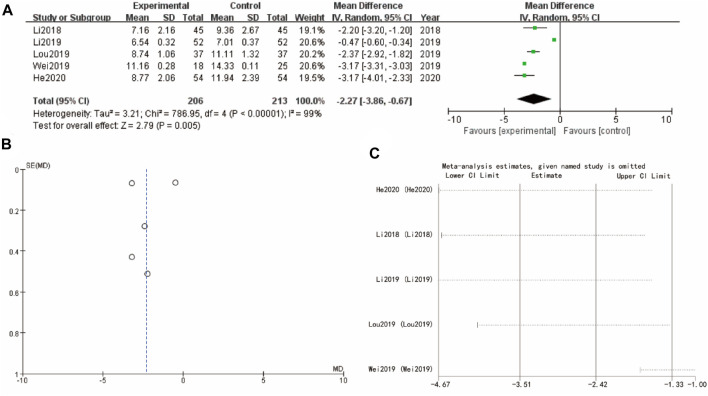
Luteinizing hormone of Yulinzhu treatment **(A)** forest plot of luteinizing hormone **(B)** funnel plot of luteinizing hormone **(C)** Sensitivity analysis.

Figure 7A’s forest plot examines luteinizing hormone with WMD = −2.27, 95% CI [–3.86, –0.67] and a *p*-value of 0.005, indicating a significant benefit for the experimental group. The funnel plot in Figure 7B shows a symmetrical distribution, suggesting minimal publication bias. Sensitivity analysis show that the differences between groups are very small (Figure 7C).

### 3.9 Testosterone

**FIGURE 8:**
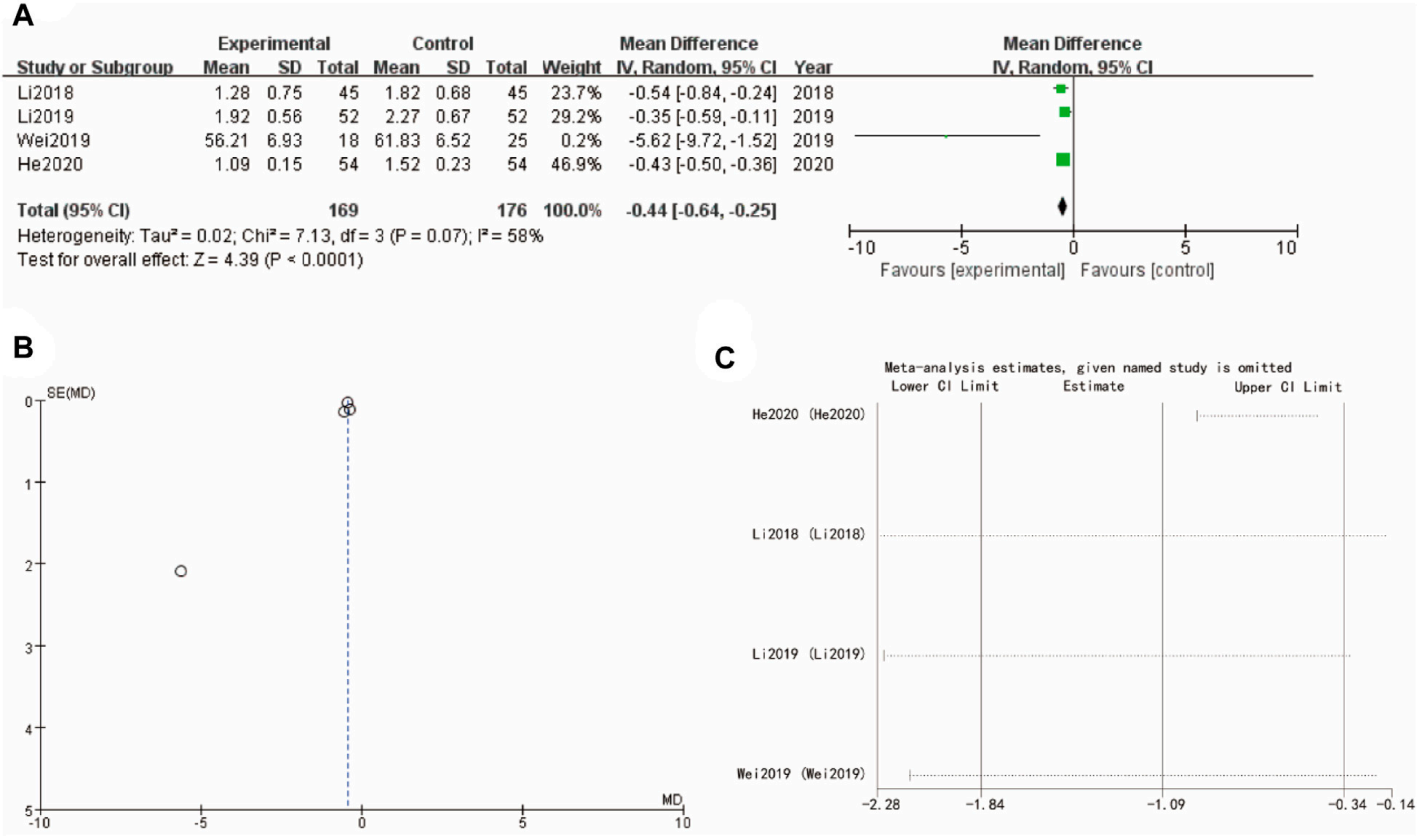
Testosterone of Yulinzhu treatment **(A)** forest plot of testosterone **(B)** funnel plot of testosterone **(C)** Sensitivity analysis.

Figure 8A of testosterone presents a forest plot with WMD = −0.44, 95% CI [−0.61, −0.25] and a highly significant p-value of less than 0.0001, reflecting a favorable effect of the experimental intervention. The funnel plot in Figure 8B indicates minimal asymmetry, implying low risk of publication bias. Sensitivity analysis show that the differences between groups are very small (Figure 8C).

### 3.10 Estradiol

**FIGURE 9:**
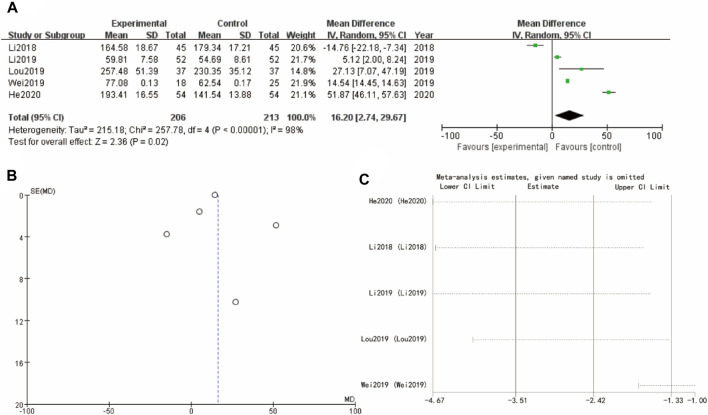
Estradiol of Yulinzhu treatment **(A)** forest plot of estradiol **(B)** funnel plot of estradiol **(C)** Sensitivity analysis.

Finally, Figure 9A of estradiol forest plot shows a WMD = 16.02, 95%CI [4.74, 26.67] with a p-value of 0.02, suggesting a significant positive effect of the experimental intervention. The corresponding funnel plot in Figure 9B is more symmetrical, indicating a low likelihood of publication bias. Sensitivity analysis show that the differences between groups are very small (Figure 9C)

### 3.11 Safety outcomes

The safety assessment of Yulinzhu was conducted based on the adverse events reported in the included studies. The most commonly reported adverse events included gastrointestinal discomfort, mild headache, and transient dizziness. These were generally mild and resolved without the need for additional treatment. No serious adverse events were reported in any of the studies. All reported adverse events were classified as mild to moderate in severity. The incidence of adverse events in the Yulinzhu treatment groups was comparable to or lower than that in the control groups, indicating a favorable safety profile.

## 4 Discussion

The findings of this study provide significant insights into the therapeutic potential of Yulinzhu, a traditional Chinese medicine (TCM) formulation, in treating Polycystic Ovary Syndrome (PCOS) induced by neurological disorders. PCOS is a multifaceted endocrine disorder that not only impacts reproductive health but also poses significant metabolic and psychological challenges. The complex interplay between the central nervous system (CNS) and ovarian function, highlighted by recent research, underscores the necessity for a holistic treatment approach that addresses both hormonal and neurological metabolites (Gao et al., 2023). We screened 891 records and included 6 studies in the meta-analysis. The results revealed that Yulinzhu had a significant effect on the effective rate (RR = 1.19, 95% CI [1.10, 1.29], *p* < 0.0001), pregnancy rate (RR = 2.80, 95% CI [1.65, 4.76], *p* < 0.0001), and ovulation rate (RR = 1.33, 95% CI [1.10, 1.62], *p* = 0.04). Additionally, the meta-analysis showed the following results: follicle estrogen (WMD = 0.69, 95% CI [-0.39, 1.78], *p* = 0.21), luteinizing hormone (WMD = −2.27, 95% CI [-3.86, −0.67], *p* = 0.005), testosterone (WMD = −0.44, 95% CI [-0.64, −0.25], *p* < 0.0001), and estradiol (WMD = 16.20, 95% CI [2.74, 29.67], *p* < 0.0001).

Our meta-analysis consolidates evidence from multiple clinical studies, demonstrating that Yulinzhu significantly improves menstrual regularity and reduces androgen levels in PCOS patients (Shang et al., 2020; Tong et al., 2022). These findings are critical, as they suggest that Yulinzhu can effectively manage the primary symptoms of PCOS, which are often resistant to conventional treatments (Ma and Tan, 2017; Zhang N. et al., 2023).

The traditional use of Yulinzhu in TCM emphasizes the harmonization of internal energies and the balance of bodily systems, which aligns well with the multifactorial nature of PCOS (Shang et al., 2021). The integration of such holistic approaches in modern medicine can offer complementary benefits to conventional treatments, particularly for conditions with complex etiologies like PCOS. The findings from this study support the potential of Yulinzhu as an effective treatment for PCOS, especially when the condition is exacerbated by neurological disorders (Ding et al., 2021). This highlights the importance of a multidisciplinary approach in managing PCOS, involving collaboration between neurologists, endocrinologists, and gynecologists (Wu et al., 2022).

The results also underscore the need for further research to fully elucidate the mechanisms underlying Yulinzhu’s effects (Teede et al., 2023). While the meta-analysis demonstrated significant benefits of Yulinzhu in treating PCOS, it is important to note the heterogeneity observed in intervention protocols and outcome measurements across the included studies. We attempted subgroup analyses based on age, treatment duration, and other factors, but these did not reduce the heterogeneity. Consequently, we performed a sensitivity analysis and found that no single study significantly affected the overall results. The observed heterogeneity in our study may arise from inherent differences in the clinical research process. To improve the generalizability and comparability of results in future research, it is essential to standardize intervention protocols and measurement indicators. Consistent application of protocols and uniform outcome measures will enhance the reliability of meta-analytic findings (Hu et al., 2021). While our meta-analysis indicates that most studies have a low risk of bias, we identified a high risk of bias in allocation concealment in individual studies such as Li 2018. This highlights the necessity for future research to adopt stringent methodological standards, including proper randomization, allocation concealment, and blinding procedures, to minimize biases and enhance the credibility of study results. Detailed descriptions of the intervention protocols and outcome measures are provided to enhance transparency and facilitate the assessment of the generalizability of the results. While the total number of participants across the included studies is indeed less than 900, we believe that the sample size is sufficient to detect significant effects and provide preliminary evidence for the efficacy of Yulinzhu in treating PCOS induced by neurological disorders. Future studies should focus on larger, well-designed clinical trials to confirm the efficacy and safety of Yulinzhu in diverse patient populations (Ye et al., 2021). Furthermore, the role of chronic stress and its impact on the hypothalamic-pituitary-gonadal (HPG) axis in PCOS development warrants further investigation. Stress-related alterations in GnRH secretion can lead to dysregulation of LH and FSH, contributing to the hormonal imbalances characteristic of PCOS. Understanding how Yulinzhu modulates these neuroendocrine pathways could enhance its therapeutic application and inform the development of more targeted treatments (Zhang Y. et al., 2023; Xu et al., 2023).

In addition to demonstrating the efficacy and safety of Yulinzhu in treating PCOS associated with neurological disorders, this study underscores the need for larger, multi-center clinical trials to confirm the findings across diverse populations. The observed heterogeneity in intervention protocols and outcome measures highlights the necessity for standardizing these elements in future research. Furthermore, while our study provides evidence for the clinical efficacy of Yulinzhu, further mechanistic studies are needed to elucidate the molecular and biochemical pathways through which Yulinzhu exerts its effects. Investigating the long-term safety and efficacy of Yulinzhu, including potential cumulative effects and the sustainability of its benefits, is also essential. Future research should explore the integration of Yulinzhu with conventional treatments for PCOS to assess potential synergistic effects and interactions. Additionally, given the multifaceted nature of Yulinzhu, its potential applications in other endocrine and neurological disorders warrant investigation. Overall, our study not only validates the traditional use of Yulinzhu in managing PCOS but also sets the stage for future research to expand and deepen our understanding of its therapeutic potential, contributing to the development of integrative treatment strategies for complex disorders.

In conclusion, this study demonstrates that Yulinzhu effectively treats PCOS associated with neurological disorders by targeting multiple pathways, including hormonal regulation, anti-inflammatory actions, and neuroprotection. These findings provide a scientific basis for the traditional use of Yulinzhu in managing PCOS and suggest potential new therapeutic strategies for addressing the neurological aspects of this disorder (Welt, 2021). The integrative approach combining traditional and modern medical practices can offer a comprehensive treatment strategy, addressing the multifaceted nature of PCOS and improving patient outcomes (Xu et al., 2022). The promising results from this study pave the way for further research into the potential of TCM formulations like Yulinzhu in treating complex endocrine disorders, highlighting the value of integrative medicine in achieving holistic health and wellbeing (Ye et al., 2021).

## Data Availability

The original contributions presented in the study are included in the article/Supplementary Material, further inquiries can be directed to the corresponding authors.
