# Supplementary material for: Plant medicine metabolite Yulinzhu treating neurological disorder causing polycystic ovary syndrome: a systematic review and a meta-analysis
Source: Front Pharmacol. 2024 Aug 15;15:1458621. doi: 10.3389/fphar.2024.1458621 (PMC11357959; doi:10.3389/fphar.2024.1458621)
Supplement: Supplementary file 2 [file Table1.DOCX]

### MEDLINE search strategy

1 Polycystic Ovary Syndrome/

2 (polycystic adj5 ovar$).tw.

3 PCOS.tw.
4 PCOD.tw.
5 (stein‐leventhal or leventhal).tw.
6 (ovar$ adj (scelerocystic or polycystic or degeneration)).tw.
7 or/1‐6

8 Yulinzhu/

9 Chinese Medcine/

10 Chinese herb/

11 or/8-10

12 randomized controlled trial.pt.
13 controlled clinical trial.pt.
14 randomized.ab.
15 placebo.tw.
16 clinical trials as topic.sh.
17 randomly.ab.
18 trial.ti.
19 (crossover or cross‐over or cross over).tw.

20 or/12-19

21 7 and 11 and 20
